# Alzheimer’s disease biomarker profiling in a memory clinic cohort without common comorbidities

**DOI:** 10.1093/braincomms/fcad228

**Published:** 2023-08-25

**Authors:** Makrina Daniilidou, Francesca Eroli, Vilma Alanko, Julen Goikolea, Maria Latorre-Leal, Patricia Rodriguez-Rodriguez, William J Griffiths, Yuqin Wang, Manuela Pacciarini, Ann Brinkmalm, Henrik Zetterberg, Kaj Blennow, Anna Rosenberg, Nenad Bogdanovic, Bengt Winblad, Miia Kivipelto, Delphine Ibghi, Angel Cedazo-Minguez, Silvia Maioli, Anna Matton

**Affiliations:** Division of Neurogeriatrics, Centre for Alzheimer Research, Department of Neurobiology, Care Sciences and Society, Karolinska Institutet, 171 64 Solna, Sweden; Division of Clinical Geriatrics, Centre for Alzheimer Research, Department of Neurobiology, Care Sciences and Society, Karolinska Institutet, 141 83 Huddinge, Sweden; Division of Neurogeriatrics, Centre for Alzheimer Research, Department of Neurobiology, Care Sciences and Society, Karolinska Institutet, 171 64 Solna, Sweden; Division of Neurogeriatrics, Centre for Alzheimer Research, Department of Neurobiology, Care Sciences and Society, Karolinska Institutet, 171 64 Solna, Sweden; Division of Clinical Geriatrics, Centre for Alzheimer Research, Department of Neurobiology, Care Sciences and Society, Karolinska Institutet, 141 83 Huddinge, Sweden; Division of Neurogeriatrics, Centre for Alzheimer Research, Department of Neurobiology, Care Sciences and Society, Karolinska Institutet, 171 64 Solna, Sweden; Division of Neurogeriatrics, Centre for Alzheimer Research, Department of Neurobiology, Care Sciences and Society, Karolinska Institutet, 171 64 Solna, Sweden; Division of Neurogeriatrics, Centre for Alzheimer Research, Department of Neurobiology, Care Sciences and Society, Karolinska Institutet, 171 64 Solna, Sweden; Swansea University Medical School, Swansea SA2 8PP, UK; Swansea University Medical School, Swansea SA2 8PP, UK; Swansea University Medical School, Swansea SA2 8PP, UK; Department of Psychiatry and Neurochemistry, Institute of Neuroscience and Physiology, Sahlgrenska Academy, University of Gothenburg, 413 90 Mölndal, Sweden; Clinical Neurochemistry Laboratory, Sahlgrenska University Hospital, 413 90 Mölndal, Sweden; Department of Psychiatry and Neurochemistry, Institute of Neuroscience and Physiology, Sahlgrenska Academy, University of Gothenburg, 413 90 Mölndal, Sweden; Clinical Neurochemistry Laboratory, Sahlgrenska University Hospital, 413 90 Mölndal, Sweden; Department of Neurodegenerative Disease, UCL Institute of Neurology, London WC1N3AR, UK; UK Dementia Research Institute at UCL, London WC1N3AR, UK; Hong Kong Center for Neurodegenerative Diseases, Clear Water Bay, Hong Kong, China; Department of Psychiatry and Neurochemistry, Institute of Neuroscience and Physiology, Sahlgrenska Academy, University of Gothenburg, 413 90 Mölndal, Sweden; Clinical Neurochemistry Laboratory, Sahlgrenska University Hospital, 413 90 Mölndal, Sweden; Department of Neurology, Institute of Clinical Medicine, University of Eastern Finland, FI-70029 Kuopio, Finland; Theme Inflammation and Aging, Karolinska University Hospital, 141 83 Huddinge, Sweden; Division of Neurogeriatrics, Centre for Alzheimer Research, Department of Neurobiology, Care Sciences and Society, Karolinska Institutet, 171 64 Solna, Sweden; Theme Inflammation and Aging, Karolinska University Hospital, 141 83 Huddinge, Sweden; Division of Clinical Geriatrics, Centre for Alzheimer Research, Department of Neurobiology, Care Sciences and Society, Karolinska Institutet, 141 83 Huddinge, Sweden; Theme Inflammation and Aging, Karolinska University Hospital, 141 83 Huddinge, Sweden; Ageing Epidemiology Research Unit, School of Public Health, Imperial College London, London SW7 2AZ, UK; Neurodegeneration Cluster, Rare and Neurologic Disease Research Sanofi R&D, F-91380 Chilly-Mazarin, France; Division of Neurogeriatrics, Centre for Alzheimer Research, Department of Neurobiology, Care Sciences and Society, Karolinska Institutet, 171 64 Solna, Sweden; Neurodegeneration Cluster, Rare and Neurologic Disease Research Sanofi R&D, F-91380 Chilly-Mazarin, France; Division of Neurogeriatrics, Centre for Alzheimer Research, Department of Neurobiology, Care Sciences and Society, Karolinska Institutet, 171 64 Solna, Sweden; Division of Neurogeriatrics, Centre for Alzheimer Research, Department of Neurobiology, Care Sciences and Society, Karolinska Institutet, 171 64 Solna, Sweden; Division of Clinical Geriatrics, Centre for Alzheimer Research, Department of Neurobiology, Care Sciences and Society, Karolinska Institutet, 141 83 Huddinge, Sweden; Ageing Epidemiology Research Unit, School of Public Health, Imperial College London, London SW7 2AZ, UK

**Keywords:** Alzheimer’s disease, biomarkers, metabolic disorders, clustering, cerebrospinal fluid

## Abstract

Alzheimer’s disease is a multifactorial disorder with large heterogeneity. Comorbidities such as hypertension, hypercholesterolaemia and diabetes are known contributors to disease progression. However, less is known about their mechanistic contribution to Alzheimer’s pathology and neurodegeneration. The aim of this study was to investigate the relationship of several biomarkers related to risk mechanisms in Alzheimer’s disease with the well-established Alzheimer’s disease markers in a memory clinic population without common comorbidities. We investigated 13 molecular markers representing key mechanisms underlying Alzheimer’s disease pathogenesis in CSF from memory clinic patients without diagnosed hypertension, hypercholesterolaemia or diabetes nor other neurodegenerative disorders. An analysis of covariance was used to compare biomarker levels between clinical groups. Associations were analysed by linear regression. Two-step cluster analysis was used to determine patient clusters. Two key markers were analysed by immunofluorescence staining in the hippocampus of non-demented control and Alzheimer’s disease individuals. CSF samples from a total of 90 participants were included in this study: 30 from patients with subjective cognitive decline (age 62.4 ± 4.38, female 60%), 30 with mild cognitive impairment (age 65.6 ± 7.48, female 50%) and 30 with Alzheimer’s disease (age 68.2 ± 7.86, female 50%). Angiotensinogen, thioredoxin-1 and interleukin-15 had the most prominent associations with Alzheimer’s disease pathology, synaptic and axonal damage markers. Synaptosomal-associated protein 25 kDa and neurofilament light chain were increased in mild cognitive impairment and Alzheimer’s disease patients. Grouping biomarkers by biological function showed that inflammatory and survival components were associated with Alzheimer’s disease pathology, synaptic dysfunction and axonal damage. Moreover, a vascular/metabolic component was associated with synaptic dysfunction. In the data-driven analysis, two patient clusters were identified: Cluster 1 had increased CSF markers of oxidative stress, vascular pathology and neuroinflammation and was characterized by elevated synaptic and axonal damage, compared with Cluster 2. Clinical groups were evenly distributed between the clusters. An analysis of post-mortem hippocampal tissue showed that compared with non-demented controls, angiotensinogen staining was higher in Alzheimer’s disease and co-localized with phosphorylated-tau. The identification of biomarker-driven endophenotypes in cognitive disorder patients further highlights the biological heterogeneity of Alzheimer’s disease and the importance of tailored prevention and treatment strategies.

## Teaser

Daniilidou *et al*. showed that several biomarkers related to risk mechanisms in Alzheimer’s disease are associated with the well-established Alzheimer’s disease markers in a memory clinic population without common comorbidities. They further defined two biologically distinct patient clusters that are likely affected by different mechanisms leading to dementia.

## Graphical Abstract

**Graphical Abstract:**
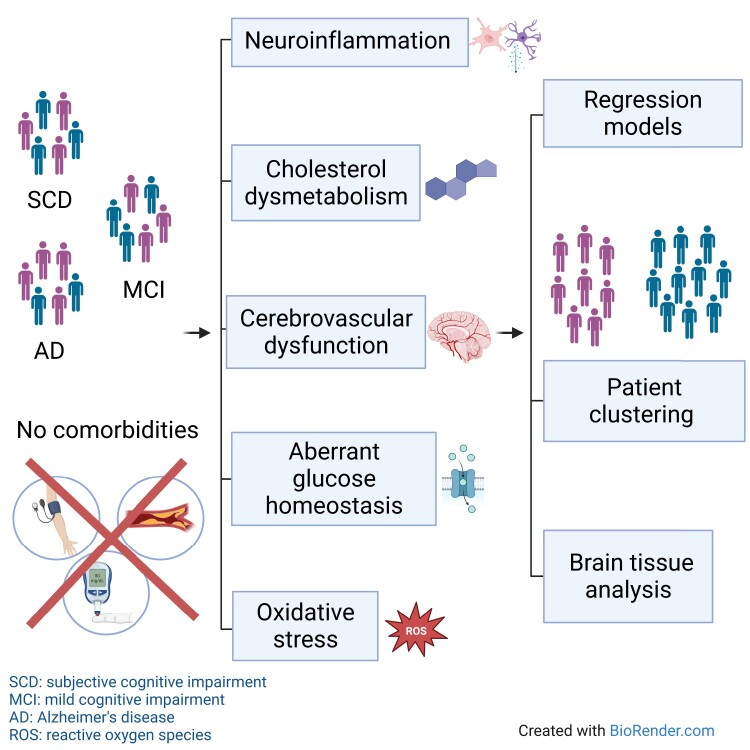

## Introduction

Alzheimer’s disease (AD) is a heterogeneous disorder considering its clinical symptoms, rate of progression, neuropathological profiles and biomarkers.^1^ The factors accounting for this heterogeneity are multiple, including age-at-onset, apolipoprotein E genotype (*APOE*) and other risk genes, lifestyle factors and comorbidities. The National Institute on Aging-Alzheimer’s Association research framework suggested a biological definition of Alzheimer’s disease that is built on biomarkers of β-amyloid, tau and neurodegeneration aiming for more harmonized cohort studies.^2^ Development of efficient and personalized treatments relies on an in-depth characterization of Alzheimer’s disease heterogeneity.^3^ As a complement to β-amyloid and tau markers, studies of additional cerebrospinal fluid (CSF) biomarkers provide information on co-existing, inducing and/or interacting mechanisms in Alzheimer’s disease. Identifying additional fluid biomarkers has, therefore, the potential to build a toolbox to stratify patient groups for mechanism-targeted treatment approaches.

For this study, in addition to core CSF Alzheimer’s disease pathology markers, 13 biomarkers reflecting different mechanisms relevant to brain health were included. Three of these biomarkers are synaptic proteins: synaptosomal-associated protein 25 kDa (SNAP-25), synaptotagmin 1 (SYT-1) and neurogranin (NG). Both SNAP-25 and SYT-1 are implicated in presynaptic neurotransmitter release, whereas NG is a postsynaptic protein involved in the calcium signalling pathway via calmodulin.^4^ Increased CSF levels of these proteins indicate synaptic dysfunction or loss and have been observed in Alzheimer’s disease^4^ and preclinical stages.^5^ Neurofilament light chain (NFL) is a subunit of the neurofilament protein found in the central and peripheral nervous system.^6^ Its concentration in CSF increases in Alzheimer’s disease and other neurodegenerative disorders and it has been proposed to represent a general biomarker of axonal injury.^6^ Markers of inflammatory processes, shown to be altered in Alzheimer’s disease, were also included in the study: the heterodimeric interleukin-12/23p40 (IL-12/IL-23p40) produced, e.g. by microglia, which influences pro-inflammatory pathways in the brain,^7^ astrocytic IL-15 that contributes to tissue damage in both neurodegeneration and acute brain injury^8^ and calprotectin (S100A8/A9) that is an inflammatory mediator produced by glial cells and implicated in Aβ pathology.^9^ The antioxidant thioredoxin-1 (TRX-1) and its cleaved form, thioredoxin-80 (TRX-80), were also assessed. Both forms have been found to protect from Aβ-induced toxicity,^10,11^ whereas TRX-80 is depleted in Alzheimer’s disease brains and CSF.^12^ There are inconsistent data on whether brain TRX-1 levels are affected.^10,13^

Insulin resistance has been shown to increase the risk of developing Alzheimer’s disease.^14^ We measured ectonucleotide pyrophosphatase/phosphodiesterase 2 (ENPP-2) (also known as autotaxin), since it has been proposed that increased CSF levels of ENPP-2 in Alzheimer’s disease reflect aberrant brain glucose homeostasis.^15^ ENPP-2 mechanism of action is to produce the bioactive lipid lysophosphatidic acid that exerts various functions in many tissues as well as in the central nervous system.^16^ To assess neurovascular function, we analysed vascular endothelial growth factor (VEGF) and angiotensinogen (AGT). VEGF is mostly known for its involvement in angiogenesis, and besides participating in brain vasculature regulation, it influences neurogenesis and neuronal regeneration.^17^ In Alzheimer’s disease, upregulation of VEGF might reflect attempts to compensate for a dysfunctional vasculature.^17^ Conversion of AGT to Angiotensin I by renin is one of the first steps in the renin–angiotensin system (RAS). In the brain, AGT is mainly produced by astrocytes.^18^ CSF AGT has been shown to be elevated in Alzheimer’s disease, as a result of an upregulated RAS.^18^ Finally, we measured CSF levels of 27-hydroxycholesterol (27-OH), a cholesterol metabolite with negative effects on neurons and inflammation.^19^ Increased 27-OH has been previously linked to memory deficits, Alzheimer’s disease and other neurodegenerative conditions.^20^

The aim of this study was to investigate CSF levels of markers reflecting brain changes in synaptic integrity, inflammation, oxidative stress, altered glucose homeostasis and cholesterol dysmetabolism in memory clinic patient groups without other neurodegenerative diseases or known Alzheimer’s disease comorbidities such as hypertension, hypercholesterolaemia and diabetes. In this population with *a priori* reduced Alzheimer’s disease risk, we wanted to explore whether these pleiotropic markers, alone or in concert with each other, interact with CSF biomarkers for Alzheimer’s disease, synaptic degeneration and memory. Samples from memory clinic patients with subjective cognitive decline (SCD), mild cognitive impairment (MCI) and Alzheimer’s disease were analysed. Linear regression models and cluster analysis were performed. Two key biomarkers of the clusters (TRX-1 and AGT) were further analysed by immunofluorescence staining of human control and Alzheimer’s disease post-mortem hippocampal tissue.

## Materials and methods

### GEDOC memory clinic subcohort

This study included 90 patients (30 subjective cognitive decline, 30 mild cognitive impairment and 30 probable Alzheimer’s disease) diagnosed at the Karolinska University Hospital memory clinic in Huddinge, Sweden, during 2008–14. Their demographical characteristics are given in Table 1. The clinical assessment at the memory clinic has been described in detail elsewhere.^21^ Briefly, it consists of a physical and neurological examination, thorough review of medical history, Mini-Mental State Examination (MMSE) and comprehensive neuropsychological testing, routine blood tests, brain imaging (MRI or CT) and CSF sampling to measure Alzheimer’s disease biomarkers (Aβ42, t-tau and p-tau). Diagnoses were made on average within 2 months from the beginning of the assessment period by consensus in multidisciplinary meetings of the clinic staff using all available clinical data, including CSF biomarker data, in an unblinded manner. Mild cognitive impairment was diagnosed using the consensus criteria for mild cognitive impairment, which require the presence of both subjective and objective cognitive impairments involving one or more cognitive domains, but no impairment of activities of daily living and no dementia.^22^ Objective cognitive impairment was defined as a test performance of 1.5 standard deviation (SD) below what is expected based on age and education. Dementia diagnoses were made according to the Diagnostic and Statistical Manual of Mental Disorders criteria,^23^ and Alzheimer’s disease aetiology was diagnosed using the National Institute of Neurological and Communicative Disorders and Stroke–Alzheimer’s Disease and Related Disorders Association criteria by McKhann *et al*.^24^ Patients who did not meet the criteria for mild cognitive impairment or dementia were considered to experience subjective cognitive decline. For this study, exclusion criteria were the presence of stroke, or other neurodegenerative disorder, hypertension, hypercholesterolaemia and diabetes.

**Table 1:** Summary of the CSF study population characteristics

Demographics	SCD (*n* = 30)	MCI (*n* = 30)	AD (*n* = 30)	*P-*value
Age, years	62.4 (4.38)	65.6 (7.48)	68.2 (7.86)	0.005
Female	60%	50%	50%	0.668
*APOE* e4 carrier^a^	31%	71%	76%	0.077
*Cognition*				
MMSE score^b^	28.1 (2.0)	27.7 (2.1)	23.2 (5.6)	<0.0001
*Core AD CSF biomarkers*				
Aβ42, pg/mL	988.5 (217.6)	569.2 (60.7)	555.3 (128.7)	<0.0001
t-tau, pg/mL	251.1 (95.1)	464.3 (219.8)	551.3 (215.1)	<0.0001
p-tau, pg/mL	61.4 (27.9)	68.4 (23.3)	74.0 (21.4)	0.108
p-tau/Aβ42	0.06 (0.03)	0.12 (0.05)	0.14 (0.05)	<0.0001
*Chronic conditions*				
Cardiovascular^c^	3.3%	6.7%	0%	0.355
Autoimmune^d^	3.3%	6.7%	6.7%	0.809
Osteoarthritis	13.3%	13.3%	6.7%	0.638

Data are shown as unadjusted mean (SD), unless otherwise stated. *P*-values were calculated by ANCOVA, adjusting for age for continuous variables or by *χ*^2^ for categorical data.

a *APOE* e4 carrier data were available for 13 SCD, 17 MCI and 17 AD participants.

b MMSE score was available for 29 SCD, 29 MCI and 26 AD participants.

c Coronary heart disease in one SCD and two MCI participants.

d Autoimmune conditions found in the cohort included psoriasis in one SCD and two MCI participants, Crohn’s disease and ulcerative colitis in two AD participants, respectively.

### Human brain tissue

Human hippocampal samples of six Alzheimer’s disease and six non-demented controls were obtained from The Netherlands Brain Bank, Netherlands Institute for Neuroscience in Amsterdam. All Alzheimer’s disease subjects met the criteria for definitive Alzheimer’s disease according to the Consortium to Establish a Registry for Alzheimer’s disease.^25^ The non-demented control subjects had no known psychiatric or neurological disorders. None of the donors had been using medication for diabetes, hypercholesterolaemia or hypertension at the time of death.

### Ethical statement

The research conducted was approved by the Ethical Review Board in Sweden (ethical permits: 2019-06056 and 2011/1987-31/4) and is in concordance with the 1964 Helsinki declaration. All participants provided their written informed consent for this study. The brain material was collected from donors whose written informed consent for brain autopsy and the use of material and clinical information for research purposes has been obtained by The Netherlands Brain Bank.

### CSF sampling

CSF samples were collected by lumbar puncture between the L3/L4 and the L4/L5 intervertebral spaces using a 25-gauge needle. Samples were collected in polypropylene tubes, centrifuged within 2 h and assessed for Aβ42, t-tau and p-tau 181 concentrations with a commercially available enzyme-linked immunosorbent assay (ELISA; Innogenetics, Ghent, Belgium). Additional sample aliquots were stored at −80°C, until they were further analysed.

### CSF biomarker measurements

#### AGT, ENPP-2, TRX-1, TRX-80, NG and NFL

The following commercially available ELISAs were used to determine CSF protein levels of AGT, ENPP-2 and TRX-1: [AGT (JP27412; TECAN, IBL International GmbH, Europe), ENPP-2 (DENP20; R&D Systems, Europe), TRX-1 (RAB1756; Sigma-Aldrich, Merck KGaA, Germany)]. All ELISAs were performed according to the manufacturers’ protocols with samples diluted 1:200 for AGT, 1:40 for ENPP-2 and 1:2 for TRX-1. NG and NFL were determined by ELISA, as previously described.^5,26^ TRX-80 was quantified by an in-house ELISA, as previously published.^10,27^ Samples were tested in duplicate, and the average was considered for the statistical analyses. The calculated intra- and inter-assay coefficients of variation (CV%) were <6 and <15%, respectively, for the overall ELISAs. Samples with duplicate CV above 15% were remeasured or excluded from the analyses. Absorbance was measured with a microplate reader (Tecan Life Sciences, Männedorf, Switzerland). Concentrations were calculated by interpolation from the standard curves using GraphPad Prism 9 software, through a 4PL curve fit.

#### IL-12/IL-23p40, IL-15, VEGF and calprotectin

IL-12/IL-23p40, IL-15, VEGF and calprotectin were measured using the ultrasensitive Mesoscale Discovery immunoassays (Mesoscale Diagnostics, Rockville, MD, USA). IL-12/IL-23p40, IL-15 and VEGF were selected from the preconfigured V-PLEX Human Cytokine Panel 1, and calprotectin was analysed using the R-Plex Human calprotectin antibody set, following the manufacturer’s protocol. A 2-fold dilution was applied for IL-12/IL-23p40 and IL-15, yet samples for VEGF and calprotectin were run undiluted. Each array was scanned in an MSD QuickPlex 120, and concentration data were retrieved using Discovery Workbench 4.0. Samples above the lowest limit of detection and with <20% intra-assay CV were considered for analysis. Inter-assay CV was <20% for all analytes.

#### SNAP-25, SYT-1 and 27-OH

SNAP-25 and SYT-1 were measured according to a previously established protocol.^28^ 27-OH was analysed following alkaline hydrolysis of sterol esters by liquid chromatography–mass spectrometry incorporating charge-tagging methodology, termed ‘Enzyme-Assisted Derivatization for Sterol Analysis’, as described previously.^29^

### Immunofluorescence staining

Deparaffinization and antigen retrieval of the brain tissue sections were performed as previously described.^30^ After washing with phosphate-buffered saline (PBS), sections were blocked in 0.1% Triton X-100 (Sigma-Aldrich), 2% normal goat serum (Thermo Fisher Scientific, MA, USA) and 1% bovine serum albumin (Sigma-Aldrich) in PBS for 1 h at room temperature. Sections were then incubated with primary antibodies diluted in blocking buffer overnight at +4°C. After 3 × 10 min washes in 0.1% Triton X-100 in PBS, they were incubated with secondary antibodies and DAPI (2-(4-Amidinophenyl)-1H-indole-6-carboxamidine) for 1 h at room temperature followed by 3 × 10 min washes in Triton X-100 in PBS. The following dilutions and antibodies were used: 1:100 mouse anti-phospho-tau (Thr212, Ser214) (MN1060; Thermo Fisher Scientific), 1:100 rabbit anti-TRX-1 (MA532569; Thermo Fisher Scientific), 1:100 rabbit anti-AGT (MA529010; Thermo Fisher Scientific), 1:500 Alexa Fluor fluorescently conjugated secondary goat anti-rabbit and anti-mouse antibodies (Thermo Fisher Scientific) and 1:100 DAPI (1351303; Bio-Rad, USA). To quench the autofluorescence of lipofuscin, sections were washed for 5 min in PBS and incubated with 0.1% w/w Sudan Black B (199664; Sigma-Aldrich) in 70% ethanol. Prior to mounting, sections were washed with Triton X-100 in PBS 2 × 10 min and 10 min with PBS.

### Image acquisition and quantification

Images of immunofluorescence-stained tissue sections were acquired using Zeiss LSM800 confocal microscope (Oberkochen, Germany) with ZEN 2 software (Blue, version 2.3). Conditions were the same for image acquisition of each experiment. Images were captured from CA1 regions, when applicable, using a 20× objective and quantified using ImageJ software.^31^ For co-localization analysis, first the number of p-tau+ cells was counted with the cell counter plugin, working on the single-channel image. The percentage of co-localization was calculated quantifying the number of p-tau+ cells that were showing co-staining with TRX-1 or AGT, out of the total p-tau+ cells.

### Statistical analysis

Descriptive statistics including mean, SD, frequency and percentages were calculated. Pairwise correlations were determined using Pearson’s *r* for normally distributed variables or Spearman test when variables were not normally distributed. Two-tailed unpaired *t*-test for normally distributed variables or Mann–Whitney *U*-test for non-normally distributed data was used for the comparison of the biomarkers between amyloid pathology status groups and sex. Two-tailed unpaired *t*-test was performed for the comparisons of the immunofluorescence co-staining intensities between control and Alzheimer’s disease subjects. For the rest of the analyses, zero skewness logarithmic transformations were applied to the continuous variables that were not normally distributed (all except ENPP-2, vascular/metabolic and survival component). Analysis of covariance (ANCOVA), covarying for age, was used for comparisons of continuous clinical, demographic and biomarker variables between diagnostic groups or clusters. *χ*^2^ was applied for categorical data. Separate linear regression models were performed with p-tau/Aβ42, SNAP-25, NFL or MMSE as outcome measure and each single marker or composite score as regressor (adjusting for age, sex and diagnosis). Composite *Z*-scores were generated as following: the vascular/metabolic composite was calculated as a mean of *Z*-scores for AGT, 27-OH and ENPP-2, the inflammatory composite was the mean *Z*-score of IL-12/23p40 and IL-15, and survival was the mean *Z*-score of TRX-1 and VEGF. The level of significance was set to *P* < 0.05. Analysis was performed using SPSS Statistics, version 28.0 (IBM Corp., IL, USA), Stata software, version 14 (StataCorp) and GraphPad Prism, version 9 (GraphPad Software, CA, USA). Biomarker profile clusters were identified using the two-step cluster analysis (SPSS Statistics/IBM Corp., version 28.0). In brief, this is a hybrid approach that first uses a distance measure to separate groups and then an agglomerative approach to choose the optimal subgroup model. In our material, log-likelihood distance measure was used. The optimal solution was determined by the programme automatically, based on the Bayesian Information Criterion. Biomarkers used to stratify the individuals were: AGT, 27-OH, ENPP-2, TRX-1, IL-12/23p40, IL-15 and VEGF.

## Results

### CSF study population characteristics

The demographical and clinical characteristics of the CSF cohort are presented in Table 1, stratified by diagnosis. In brief, Alzheimer’s disease participants were significantly older than subjective cognitive decline and mild cognitive impairment individuals (*P* = 0.005), whereas sex distribution did not differ between the three diagnostic groups (*P* = 0.68). In addition, MMSE score was lower in Alzheimer’s disease compared with the other two groups after age adjustment (*P* < 0.0001). CSF Aβ42 levels were significantly lower and t-tau higher in Alzheimer’s disease compared with subjective cognitive decline and mild cognitive impairment participants (*P* < 0.0001 for both comparisons), while p-tau levels did not differ after age correction (*P* = 0.108). *APOE* e4 carriers tended to be more frequent in Alzheimer’s disease patients (*P* = 0.077). There were no differences in the presence of other known chronic conditions between the groups.

### CSF levels of the biomarkers stratified by diagnostic groups and β-amyloid pathology status

**Figure 1:**
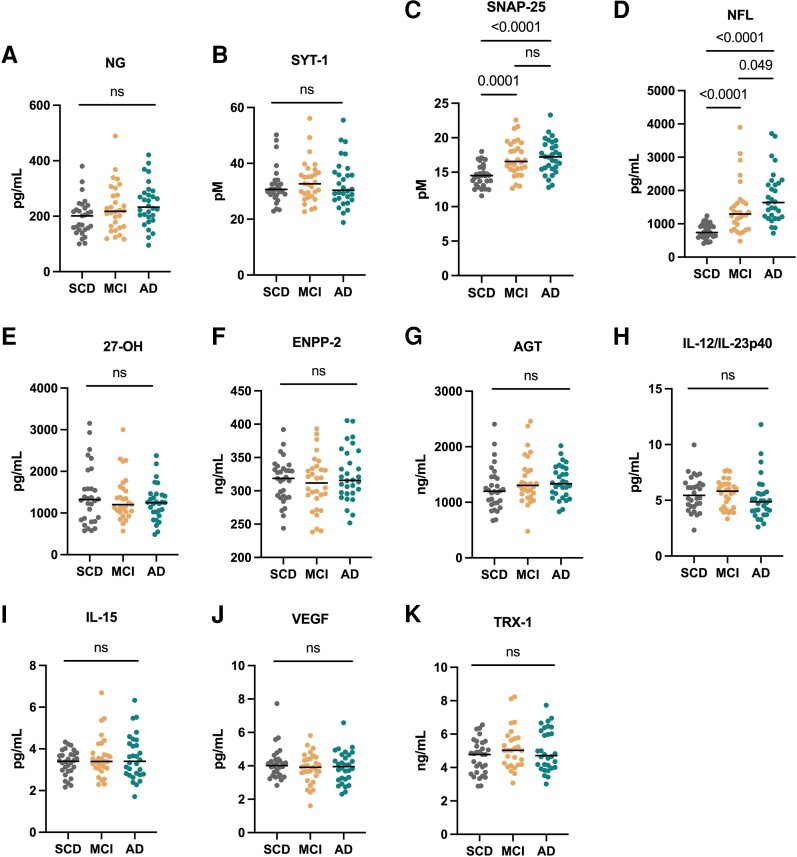
**Scatter plots depicting biomarker levels across clinical groups (A–K).** Biomarker concentrations are in *y*-axis. Median is shown as a horizontal line. *P*-values were calculated by ANCOVA, adjusting for age.

As shown in Fig. 1A–K, we initially explored CSF levels of the individual biomarkers, all stratified by diagnosis. To eliminate the possible confounding effect of age difference between the groups, all analyses were performed after age adjustment. SNAP-25 and NFL differed significantly among the three diagnostic groups. More specifically, SNAP-25 was higher both in Alzheimer’s disease and mild cognitive impairment versus subjective cognitive decline (*P* < 0.0001 and *P* = 0.0001, respectively; Fig. 1C). NFL was higher in Alzheimer’s disease compared with both mild cognitive impairment and subjective cognitive decline participants (*P* = 0.049 and *P* < 0.0001) and in mild cognitive impairment versus subjective cognitive decline (*P* < 0.0001; Fig. 1D). No other group level differences could be observed for the rest of the biomarkers.

TRX-80 and calprotectin were not detectable in a sufficient number of CSF samples and thus excluded from further analysis. From a total of 90 individuals, calprotectin was detected in 34, while TRX-80 in 31. No differences were found in the frequency of detectable/non-detectable cases among the diagnostic groups for both biomarkers (*P* = 0.231 for calprotectin and *P* = 0.640 for TRX-80) or in the levels among individuals with quantifiable data (*P* = 0.466 for calprotectin and *P* = 0.546 for TRX-80; Supplementary Fig. 1A–D).

When comparing biomarker levels by amyloid pathology status (A− for CSF Ab42 levels >550 pg/mL, A+ for CSF Ab42 levels <550 pg/mL), we found that several of the investigated markers were higher in A+ versus A− participants; NG (*P* = 0.021), SNAP-25 (*P* < 0.0001), NFL (*P* < 0.0001), 27-OH (*P* = 0.027), IL-15 (*P* < 0.001) and TRX-1 (*P* = 0.041; Supplementary Fig. 2A, C, D, E, I, K). Basic characteristics of the β-amyloid status groups are shown in Supplementary Table 1.

### CSF biomarker correlations to age, sex and one another

**Figure 2:**
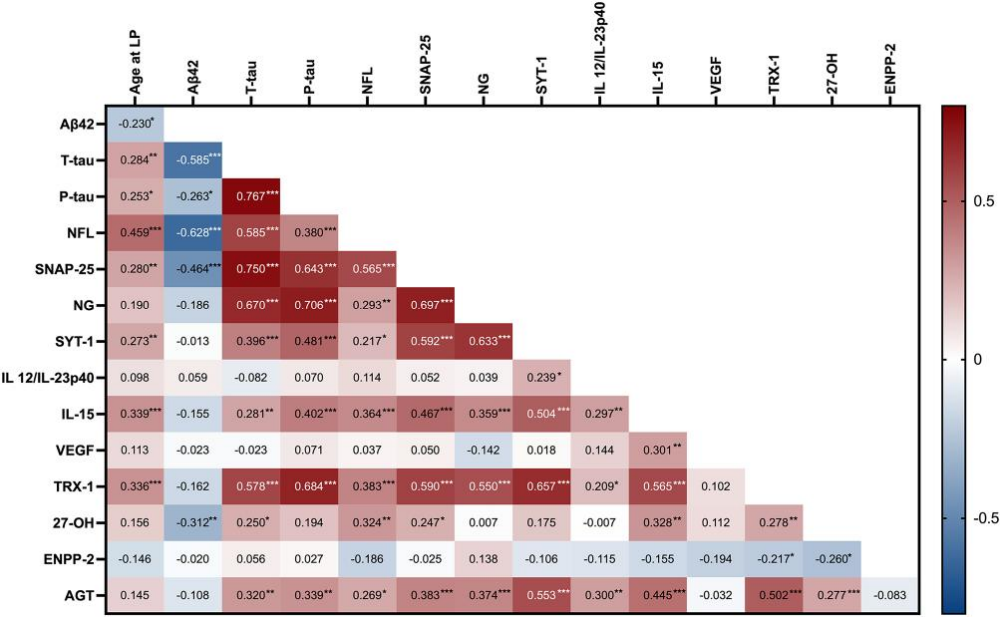
**Correlation matrix of the analysed biomarkers.** Correlation coefficients indicated in red/blue depending on the direction of the association (red: positive association, blue: negative association). Pearson test was applied for correlations between normally distributed variables, otherwise Spearman test was performed. A total of 90 samples were included in the analysis (1 value was missing for SNAP-25, NG, SYT-1, NFL and TRX-1 and 2 values for 27-OH). LP, lumbar puncture. *P < 0.05, **P < 0.01, ***P < 0.001.

We next explored the relationship of each of the markers with sex, age and one another, as well as with the established Alzheimer’s disease biomarkers (Aβ42, t-tau and p-tau). NG and IL-12/IL-23p40 were higher in women than in men (*P* = 0.037 and *P* = 0.048, respectively; Supplementary Table 2). Correlations to age and between markers are presented in a correlation map in Fig. 2. Our results show that SNAP-25, NFL, SYT-1, IL-15, TRX-1, as well as Aβ42, t-tau and p-tau, correlated all with age. Aβ42 levels correlated negatively with t-tau, p-tau, SNAP-25, NFL and 27-OH levels. Higher t-tau and p-tau were associated with higher NFL, synaptic markers, IL-15, TRX-1 and AGT. Additionally, 27-OH and t-tau correlated positively. As shown in the correlation map, IL-15, TRX-1 and AGT had the highest number of significant correlations, after the core Alzheimer’s disease and synaptic biomarkers. All three correlated with NFL, synaptic markers, IL-12/IL-23p40, 27-OH and with one another. Moreover, IL-15 correlated positively with VEGF and TRX-1 negatively with ENPP-2.

### Associations between mechanistic biomarkers and key elements of Alzheimer’s disease pathogenesis

We further explored whether the investigated biomarkers of cholesterol dysmetabolism (27-OH) vascular function (AGT, VEGF), inflammation (IL-12/IL-23p40, IL-15), oxidative stress (TRX-1) and glucose homeostasis (ENPP-2) are associated with Alzheimer’s disease pathology, synaptic dysfunction, neurodegeneration and cognition. To this end, we performed separate linear regression models with p-tau/Aβ42, SNAP-25, NFL and MMSE score as outcome variables and each of the different biological mechanism biomarkers as regressors. All models were adjusted for age, sex and diagnosis. p-tau/Aβ42 ratio was preferentially selected as a specific marker for Alzheimer’s disease instead of individual markers, as it has been shown to be superior in assessing β-amyloid pathology.^32^ SNAP-25 was the only synaptic marker that differed between the clinical groups in our material and it has been shown to possess the best discriminatory power to distinguish Alzheimer’s disease from non-Alzheimer patients compared with other synaptic biomarkers^28^; therefore, we selected it as a synaptic dysfunction outcome measure. The results of the analysis are summarized in Table 2. In agreement with our correlation findings, IL-15, TRX-1 and AGT showed the most significant associations. Specifically, IL-15 and TRX-1 were positively associated with p-tau/Aβ42 ratio (*β* = 0.300, *P* < 0.0001 and *β* = 0.451, *P* < 0.0001, respectively), with SNAP-25 (*β* = 0.507, *P* < 0.0001 and *β* = 0.593, *P* < 0.0001, respectively) and with NFL (*β* = 0.371, *P* < 0.0001 and *β* = 0.310, *P* < 0.0001, respectively). AGT was associated with SNAP-25 (*β* = 0.354, *P* < 0.0001) and NFL (*β* = 0.160, *P* = 0.047). Furthermore, 27-OH and IL-12/IL-23p40 were associated with NFL (*β* = 0.223, *P* = 0.007 and *β* = 0.260, *P* = 0.001). ENPP-2 showed a negative association with NFL (*β* = −0.162, *P* = 0.047). IL-15 was the only marker that was associated with MMSE score (*β* = 0.259, *P* = 0.015). No associations could be observed for VEGF. Adding other chronic medical conditions (cardiovascular disease, autoimmune disease or osteoarthritis) into each model separately did not change the results (data not shown).

**Table 2:** Linear regression models

	p-tau/Aβ42 *β* (*P*-value)	SNAP-25 *β* (*P*-value)	NFL *β* (*P*-value)	MMSE *β* (*P*-value)
*Single biomarkers*				
AGT	0.103 (0.202)	0.354 (<0.001)	0.160 (0.047)	0.104 (0.301)
27-OH	0.150 (0.067)	0.168 (0.092)	0.223 (0.007)	0.172 (0.090)
IL-12/IL-23p40	0.074 (0.369)	0.134 (0.173)	0.260 (0.001)	−0.003 (0.977)
IL-15	0.300 (<0.001)	0.507 (<0.0001)	0.371 (<0.0001)	0.259 (0.015)
TRX-1	0.451 (<0.0001)	0.593 (<0.0001)	0.310 (<0.001)	0.094 (0.391)
VEGF	0.128 (0.117)	0.116 (0.240)	0.067 (0.419)	0.043 (0.673)
ENPP-2	0.018 (0.829)	−0.024 (0.809)	−0.162 (0.047)	−0.148 (0.136)
*Composites*				
Vascular/metabolic	0.143 (0.080)	0.298 (0.002)	0.115 (0.164)	0.047 (0.641)
Inflammatory	0.237 (0.004)	0.374 (<0.001)	0.412 (<0.0001)	0.141 (0.180)
Survival	0.357 (<0.0001)	0.448 (<0.0001)	0.249 (0.003)	0.092 (0.390)

Results are from separate linear regression models with p-tau/Aβ42, SNAP-25, NFL or MMSE as outcome measure and each single biomarker or composite score as regressor. Data are shown as standardized *β* coefficients (*P-*values), after age, sex and diagnosis adjustment.

### Stratification of biomarkers into composite scores

**Figure 3:**
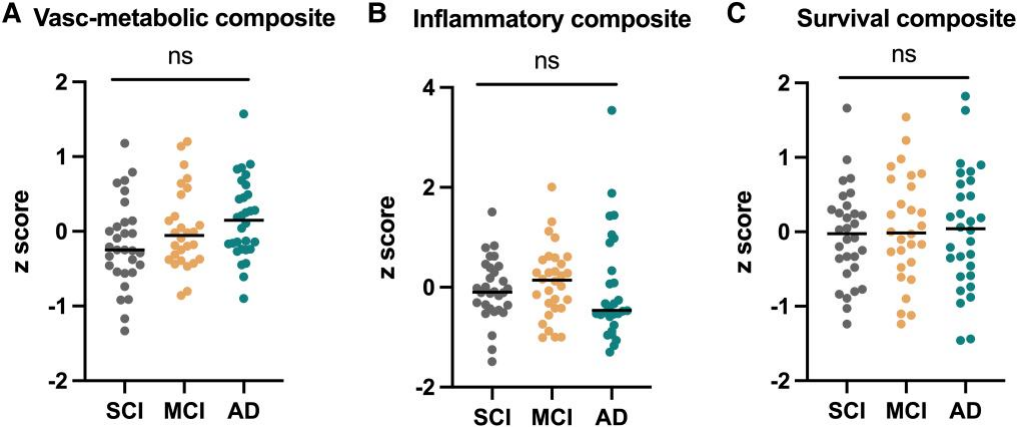
**Scatter plots of CSF biomarker composite scores in SCD, MCI and AD.** Individual-level *Z*-scores of the composites in all included subjects are plotted: (**A**) vascular/metabolic composite score, (**B**) inflammatory composite score and (**C**) survival composite score. *P*-values were calculated by ANCOVA, adjusting for age.

We next aimed to explore if, by grouping the different biomarkers into biological mechanisms, we could extract additional information and increase our understanding of contributing mechanisms to Alzheimer’s disease. We therefore sorted them based on their association with specific biological functions (for references, see Nitsch *et al*.,^7^ Janelidze *et al*.,^8^ Akterin *et al*.,^10^ Arodin *et al*.,^13^ McLimans and Willette,^15^ Herr *et al*.,^16^ Lange *et al*.,^17^ Cosarderelioglu *et al*.^18^ and Loera-Valencia *et al*.^20^), resulting into three hypothetical variables, represented as composite scores: vascular/metabolic, inflammation and survival. The vascular/metabolic composite was calculated as a mean of *Z*-scores for AGT, 27-OH and ENPP-2, the inflammatory composite was the mean *Z*-score of IL-12/23p40 and IL-15 and survival was the mean *Z*-score of TRX-1 and VEGF. The distribution of the composite scores among clinical groups is shown in Fig. 3. There were no significant differences between clinical groups for any of the composites. Stratifying the patients into amyloid positive versus negative showed an increased score for the survival component in subjects with amyloid pathology (Supplementary Fig. 3C).

Subsequently, we explored the relationship of each composite score with Alzheimer’s disease pathology (p-tau/Aβ42), synaptic dysfunction (SNAP-25), neurodegeneration (NFL) and cognition (MMSE) in linear regression models adjusting for age, sex and diagnosis (Table 2). All composite scores were associated with SNAP-25 (*β* = 0.298, *P* = 0.002 for vascular/metabolic, *β* = 0.374, *P* < 0.0001 for inflammatory and *β* = 0.448, *P* < 0.0001 for survival composite). Inflammatory and survival composites were additionally associated with p-tau/Aβ42 (*β* = 0.237, *P* = 0.004 and *β* = 0.357, *P* < 0.0001, respectively) and with NFL (*β* = 0.412, *P* < 0.0001 and *β* = 0.249, *P* = 0.003, respectively). None of them were associated with cognition. Adding other chronic medical conditions into each model separately did not change the results (data not shown).

### Patient clustering by biological mechanisms

**Figure 4:**
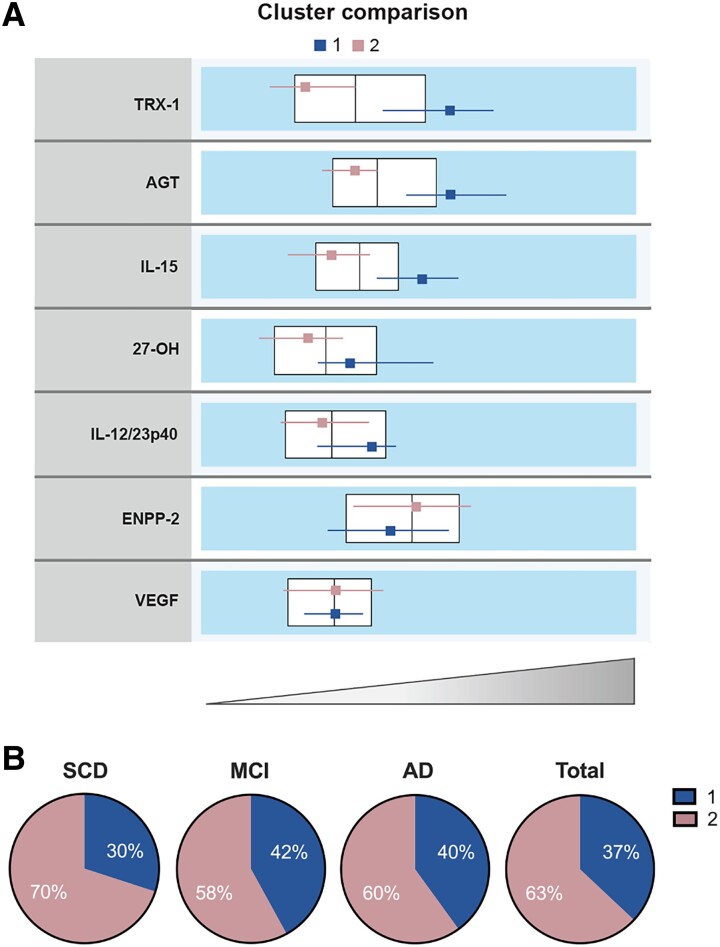
**Multivariate clustering of patients.** (**A**) Clusters comparison showing horizontal box plots of the included markers. Large horizontal boxes represent the interquartile range and vertical line the median of each biomarker in the total cohort. Small boxes depict the median and their respective lines the interquartile range for each cluster. (**B**) Percentage of patients who are distributed in Clusters 1 and 2 within each clinical diagnosis. A total of 86 participants were included in the analysis.

To assess distinct biomarker profiles within our cohort, we applied a two-step clustering analysis in the whole data set (*n* = 86, four samples were not eligible due to missing data). This data-driven unbiased clustering was based on the seven relatively unexplored biomarkers related to pathways conferring increased Alzheimer’s disease risk. Two clusters were identified (Fig. 4A), and the drivers of this stratification were mainly TRX-1, AGT and IL-15. Individuals assigned in Cluster 1 were characterized by parallel increases of CSF TRX-1 (*P* < 0.0001), AGT (*P* < 0.0001), IL-15 (*P* < 0.0001), 27-OH (*P* = 0.004) and IL-12/IL-23p40 (*P* = 0.002; Fig. 4A and Table 3) compared with Cluster 2. Cluster 1 (*n* = 32) contained 37% of the cohort population, including 30% of the subjective cognitive decline, 42% of the mild cognitive impairment and 40% of the Alzheimer’s disease groups (Fig. 4B). In comparison with Cluster 2, individuals in Cluster 1 were older (*P* < 0.0001), showed higher levels of SNAP-25 and NFL (*P* < 0.0001 and *P* = 0.011, respectively) and a tendency to increased p-tau/Aβ42 ratio (*P* = 0.080; Table 3). No differences in the levels of ENPP-2 (*P* = 0.224) and VEGF (*P* = 0.434) were seen between both clusters. Also, both clusters had similar clinical group distributions (*P* = 0.590, Pearson’s *χ*^2^).

**Table 3:** Demographic, cognition and biomarker comparisons between clusters

	Cluster 1 (*n* = 32)	Cluster 2 (*n* = 54)	*P*-value
Age	68.4 (7.1)	63.2 (5.9)	<0.0001
Female, %	62.5%	46.3%	0.146
MMSE	27.4 (2.5)	25.8 (4.9)	0.016
p-tau/Aβ42	0.12 (0.06)	0.10 (0.05)	0.080
SNAP-25, pM	17.5 (2.3)	15.3 (2.0)	<0.0001
NFL, pg/mL	1654.8 (848.8)	1123.5 (551.8)	0.011
TRX-1, ng/mL	5.9 (0.9)	4.3 (0.8)	<0.0001
AGT, ng/mL	1646.1 (373.4)	1148.7 (234.3)	<0.0001
IL-15, pg/mL	4.1 (0.8)	3.1 (0.6)	<0.0001
27-OH, pg/mL	1565.5 (0.630.6)	1196.2 (486.1)	0.004
IL-12/IL-23p40, pg/mL	6.0 (1.7)	5.1 (1.4)	0.002
ENPP-2, ng/mL	310.1 (40.4)	323.3 (34.8)	0.224
VEGF, pg/mL	3.9 (0.6)	4.0 (1.1)	0.434

Data are shown as unadjusted mean (SD), unless otherwise stated. *P*-values were calculated by ANCOVA, adjusting for age for continuous variables or by *χ*^2^ for categorical data.

Despite individuals in Cluster 1 showing increased CSF levels of synaptic biomarkers (suggesting higher neurodegeneration), their overall cognitive performance in MMSE was found higher than in Cluster 2 (*P* = 0.016; Table 3).

### Immunofluorescence staining of TRX-1 and AGT in the human brain

**Figure 5:**
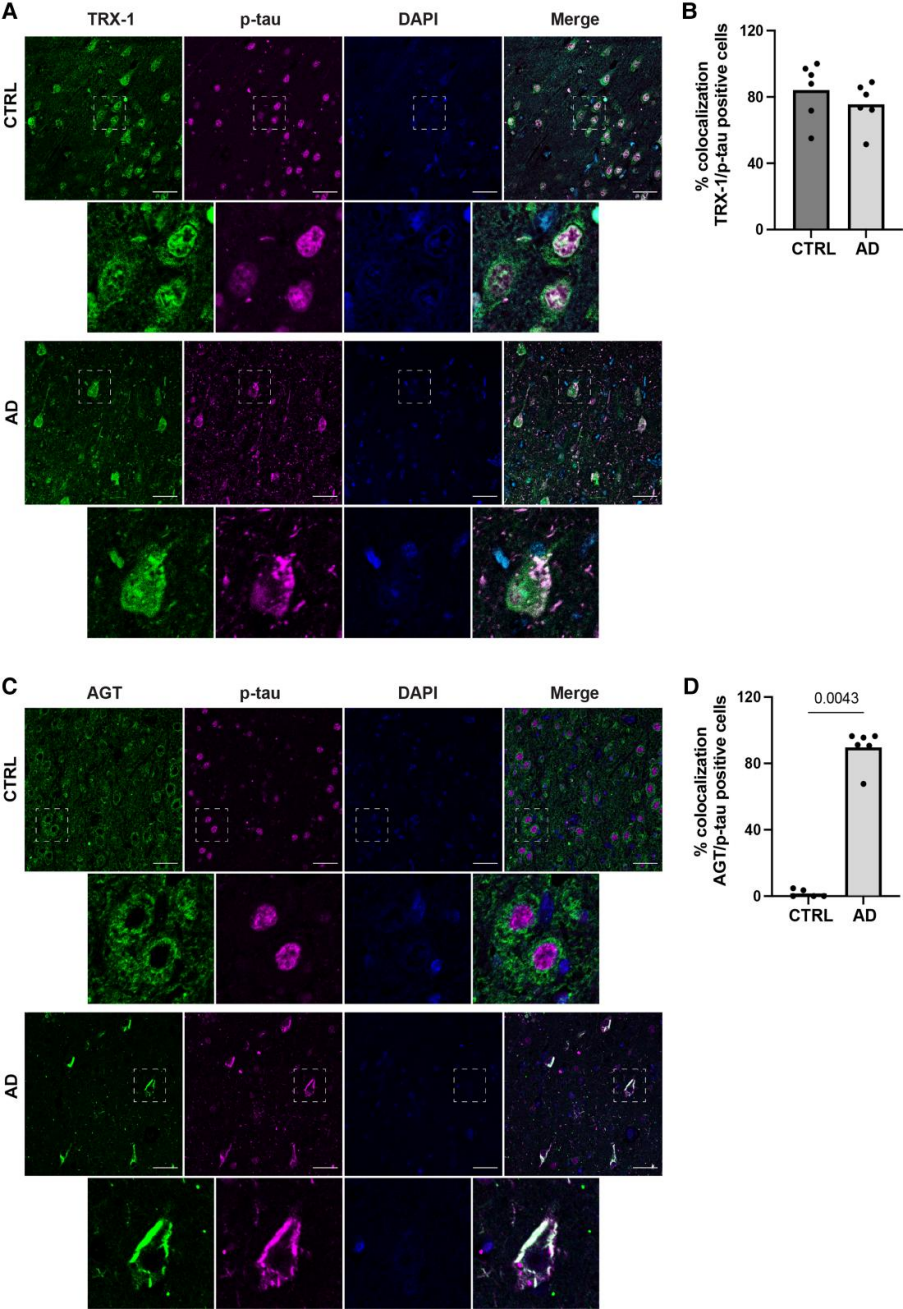
**Immunofluorescence co-staining of p-tau with thioredoxin-1 (TRX-1) or angiotensinogen (AGT) in human hippocampal sections.** (**A**) Control and AD hippocampi sections immunostained for TRX-1 (green), p-tau (red) and DAPI for nuclei (blue). (**B**) Bar charts showing mean % TRX-1/p-tau colocalization in each clinical group, with individual values in dots. (**C**) Immunofluorescent staining of AGT (green), p-tau (red) and DAPI in control (upper panels) and AD (lower panels). (**D**) Bar charts of mean % AGT/p-tau colocalization in each clinical group, with individual values in dots. P values were calculated by two-tailed unpaired t test. Respective higher magnifications (dashed line) are presented below each panel. Scale bars, 50 mm.

Since TRX-1 and AGT were two of the CSF markers with the strongest associations to neurodegenerative markers and, at the same time, relatively scarcely studied in human brain tissue, we explored their distribution and levels in hippocampal sections from Alzheimer’s disease and non-demented age-matched subjects (controls). Since p-tau was one of the markers where we found correlations to AGT and TRX-1 in the CSF, we co-stained the sections for p-tau. Supplementary Table 3 displays information about the donor characteristics. TRX-1 had a nuclear and cytoplasmic distribution in Alzheimer’s disease and control cases (Fig. 5A). We did not observe differences in TRX-1 immunofluorescence intensity between the two groups. In control brains, p-tau appeared to be localized mostly in the nuclei, while in Alzheimer’s disease, it was found mainly in the cytoplasm. TRX-1 co-localized with p-tau at a similar extent in both groups (Fig. 5A and B). Immunofluorescence labelling for AGT was more pronounced in Alzheimer’s disease than in control samples (Fig. 5C). In Alzheimer’s disease sections, there was a prominent co-staining between AGT and p-tau (*P* = 0.004; Fig. 5C and D).

## Discussion

In this study, we investigated the relationship of CSF biomarkers for Alzheimer’s disease and neurodegeneration with markers reflecting disturbances in cholesterol homeostasis, vascular function, inflammation, redox capacity and glucose homeostasis in a memory clinic cohort. Importantly, we selected individuals free of hypercholesterolaemia, hypertension or Type 2 diabetes, to eliminate as possible the contribution of these comorbidities often found in Alzheimer’s disease patients.

We found that SNAP-25 and NFL were increased in mild cognitive impairment and Alzheimer’s disease compared with the subjective cognitive decline group and as well as in patients with β-amyloid pathology. Together with SNAP-25, CSF levels of the presynaptic protein SYT-1 and the postsynaptic NG are increased in early stages of the Alzheimer’s disease continuum and correlate with cognitive decline, Aβ and tau pathology.^5,28,33,34^ In our study, NG levels were increased in A+ individuals and tended to be elevated in Alzheimer’s disease patients (though not significantly, *P* = 0.084), while SYT-1 did not vary across the three clinical groups. In agreement with our results, NFL has been previously found to be upregulated in the CSF of Alzheimer’s disease patients compared with healthy controls as well as in A+ subjects.^4,35^

Although there were no differences in the concentrations of IL-15 and 27-OH between the diagnostic groups, these biomarkers were elevated in A+ subjects. The role of neuroinflammation in Alzheimer’s disease is pivotal,^36^ and among other cytokines, IL-15 has been implicated in the disease pathology and found to be elevated in patients with pathologic β-amyloid status,^8^ as in our cohort.

Previous data on the relationship of 27-OH with β-amyloid pathology in humans are conflicting. It has been shown that CSF 27-OH correlates with sAPP but not with Ab42 levels.^37^ The increased concentration of 27-OH in A+ patients found here further suggests the link of cholesterol metabolism with Aβ production; however, the underlying mechanisms are yet to be elucidated.

TRX-1, AGT and IL-15 emerged as the biomarkers that showed the highest number of associations with the other individual biomarkers. TRX-1 is a major component of the oxidative stress response machinery.^38^ In Alzheimer’s disease, this activity is impaired, leading to an imbalance in redox homeostasis.^39^ So far, CSF TRX-1 levels have not been extensively explored in the context of Alzheimer’s disease. In an earlier study, Arodin *et al*.^13^ suggested that TRX-1 was increased in Alzheimer’s disease and in mild cognitive impairment converters. In the present study, there were no differences between the clinical groups. This could be attributed to a discrepancy of the cohorts or to methodological issues, e.g. different assays used to quantify TRX-1. However, TRX-1 was elevated in A+ individuals and was associated with p-tau/Aβ42 ratio, SNAP-25 and NFL, supporting its implication in Alzheimer’s disease pathogenesis. TRX-1 correlated also with the cytokines IL-12/IL-23p40 and IL-15 in our cohort. In line with the presented data, it has been documented that TRX-1 is involved in inflammation by inducing both pro-inflammatory and anti-inflammatory processes.^40^ In this study, TRX-1 correlated with AGT and 27-OH, suggesting that it may be implicated in diverse pathological mechanisms occurring in Alzheimer’s disease through its ubiquitous function. Immunohistochemical analyses of human hippocampal samples confirmed the absence of TRX-1 levels changes in Alzheimer’s disease individuals compared with non-demented controls. A co-localization with p-tau was evident in both groups, suggesting a more general, non-Alzheimer’s disease–specific association. A limited number of studies have explored TRX-1 distribution in the human Alzheimer’s disease brain so far, with contradictory results, as TRX-1 was found to be either decreased^10,41^ or unchanged between Alzheimer’s disease and control brains.^13^ The discrepancies between the different studies could be attributed to methodological or cohort differences, such as the selection of antibodies, or a population free of common comorbidities as in our material.

Hyperactivation of the brain RAS has been linked to Alzheimer’s disease,^42^ yet data on the levels of AGT in the human Alzheimer’s disease brain and CSF are very limited. A previous study showed AGT upregulation in Alzheimer’s brains,^19^ while others reported no alterations.^43^ However, the presence or not of mixed comorbidities in these cohorts was not evaluated. We report here that AGT was associated with NFL and SNAP-25. The effects of RAS activation in the brain are receptor dependent; activation of Angiotensin II Type 1 receptor has a largely negative impact resulting in, e.g. inflammation and oxidative stress, while activation of Angiotensin II Type 2 receptor has a protective role (as reviewed in Cosarderelioglu *et al*.^18^). Thus, the observed positive correlation between TRX-1 and AGT may reflect an interplay in the context of enhanced oxidative stress. Furthermore, we found a positive correlation between AGT and 27-OH. Previous *in vitro* and *in vivo* work have shown that 27-OH induces AGT production in the brain, implying a connection between hypercholesterolaemia and hypertension in neurodegeneration.^44^ In agreement with Mateos *et al*.,^19^ we show that AGT is increased in post-mortem hippocampal tissue of Alzheimer’s disease donors compared with controls, suggesting an altered AGT synthesis or cleavage independent of the presence of hypertension, hypercholesterolaemia or diabetes. Moreover, AGT co-localized extensively with p-tau in Alzheimer’s disease brains, further supporting its implication in the disease pathology.

Interestingly, p-tau was predominantly located in the nucleus of control brains. Although it is a cytosol-enriched protein, tau has been shown to localize in the nucleus of the mammal brain.^45,46^

We followed two distinct strategies to identify pathophysiological profiles that could reflect different cognitive disorder phenotypes, based on the current clinical diagnostic criteria. In both analyses, we included the less explored markers of Alzheimer’s disease–risk pathologies, as the traditional would have probably overpowered the analyses. In the first approach, biomarkers were grouped based on their function, generating three components, i.e. vascular/metabolic, inflammatory and survival. This categorization was challenging as many of these proteins are pleiotropic and could fit in more than one group, still they were categorized based on a meaningful biological relevance. There was no relationship between the clinical and biomarker-based groups, although a tendency towards a higher vascular/metabolic profile in the Alzheimer’s disease group was observed (albeit not statistically significant, *P* = 0.078). Thus, the amount of contribution from these biological processes was not necessarily reflected in the diagnostic categories. However, when stratifying the patients based on β-amyloid status, the survival component was increased in A+ subjects, a result possibly driven by TRX-1, which could reflect a rebounded mechanism against β-amyloid pathology. All components were associated with synaptic dysfunction, and inflammatory and survival components were associated with an Alzheimer’s disease CSF profile and axonal damage, suggesting a pathophysiological role over the disease continuum.

Using a data-driven strategy, individuals were stratified into two distinct biomarker-driven clusters. The proteins that contributed the most in the clustering were TRX-1, AGT and IL-15. Individuals in Cluster 1 were defined by higher levels of TRX-1, AGT, IL-15, 27-OH and IL-12/IL-23p40, thus showing an endophenotype characterized by increased oxidative stress, vascular and cholesterol metabolism pathology and neuroinflammation. Furthermore, Cluster 1 consisted of older participants with increased SNAP-25 and NFL compared with Cluster 2. Hence, this could mirror the contribution of these pathophysiological pathways in Alzheimer’s disease progression through a process potentially independent of β-amyloid and tau pathology. Paradoxically, Cluster 1 individuals had a better MMSE score. Interestingly, none of the two clusters was associated with a particular clinical diagnosis. Similarly, both were equally represented in each clinical group, further highlighting the biological heterogeneity observed in Alzheimer’s disease^47^ even when reducing the number of confounding comorbidities. In this context, precision medicine is the key towards a more effective treatment than the ‘one drug fits all’ concept.^48,49^ Our work provides further knowledge to assist in the implementation of a personalized treatment where individuals with specific ‘molecular’ profiles linked to Alzheimer’s disease pathogenesis could benefit from certain interventions targeting these altered processes.

The current study comes with some limitations. First, the sample size is relatively small which may explain the lack of between-group differences in most of the individual and combined markers. This may also be due to the fact that this cohort derives from a real-world memory clinic with more heterogeneity in terms of their AD CSF profiles. It should be noted though that the inclusion criteria limited substantially the number of eligible participants. Second, the study was cross-sectional, and therefore, the directionality of the relationships found here cannot be addressed. Third, a correction for multiple comparisons was not applied due to the explorative nature of this study, and therefore, the conclusions should not be generalized. A strength of the study was the selection of individuals not diagnosed or treated for common comorbidities seen in Alzheimer’s disease. Nevertheless, adding groups of individuals with comorbidities would further enhance this assumption.

## Conclusion

We defined a set of molecular markers representing key Alzheimer’s disease–risk mechanisms to be associated individually or in combinations with the well-established Alzheimer’s disease and neurodegeneration markers. As the population included in our study did not suffer from hypertension, diabetes or hypercholesterolaemia, our findings suggest that the relationships found here are independent of these peripheral pathological conditions. We identified two biologically distinct endophenotypes in memory clinic patients who are likely to be affected by different mechanisms, leading to cognitive impairment or Alzheimer’s disease. Our findings support the complex interplay of several key pathophysiological mechanisms to Alzheimer’s disease pathology and further highlight the biological heterogeneity in Alzheimer’s disease with relevant clinical applications.

## Supplementary Material

fcad228_Supplementary_Data

## Data Availability

Anonymized data will be shared with qualified investigators who have Institutional Review Board approval and a Material Transfer Agreement on request.
