# Supplementary material for: Alzheimer’s disease biomarker profiling in a memory clinic cohort without common comorbidities
Source: Brain Commun. 2023 Aug 25;5(5):fcad228. doi: 10.1093/braincomms/fcad228 (PMC10481253; doi:10.1093/braincomms/fcad228)
Supplement: fcad228_Supplementary_Data [file fcad228_supplementary_data.pdf]

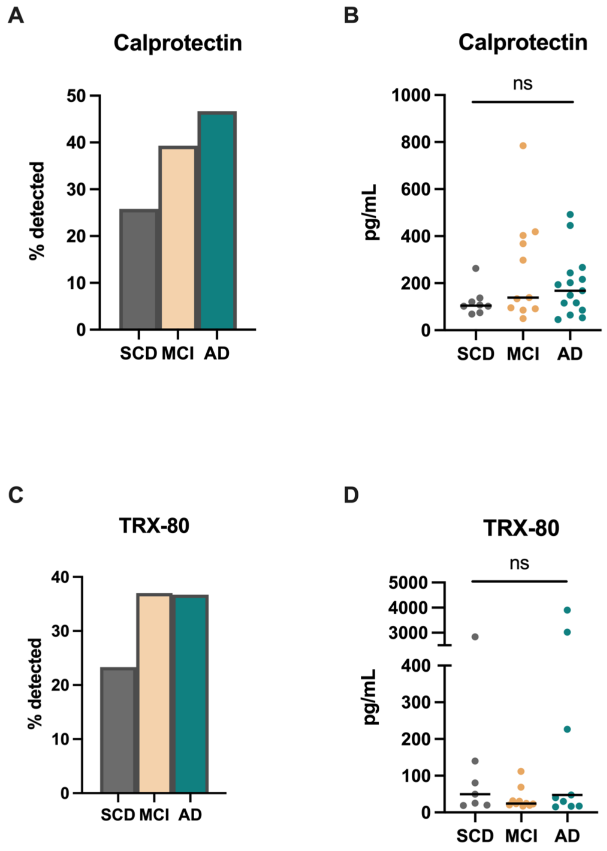

Supplementary Figure 1. Calprotectin and TRX-80 detectable cases and concentrations across clinical groups. (A) Frequency (%) of calprotectin detectable cases. **(B)** Calprotectin quantification levels in detectable cases, median shown as horizontal line. **(C)**. Frequency (%) of TRX-80 detectable cases. **(D)** TRX-80 quantification levels in detectable cases, median shown as horizontal line. *P* values were calculated by analysis of covariance (ANCOVA), adjusting for age.

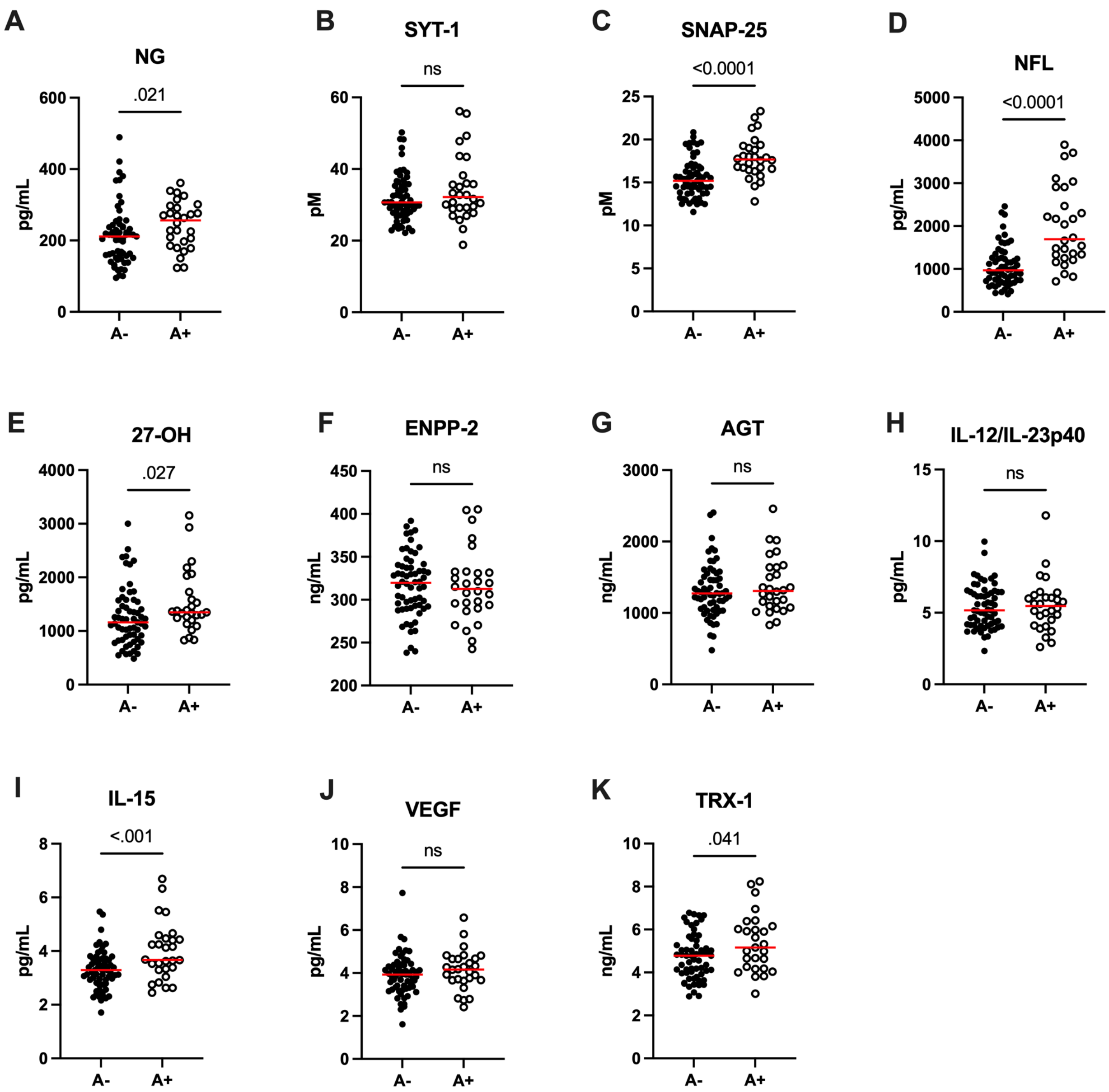

Supplementary Figure 2. Scatter-plots depicting biomarker levels across amyloid pathology status groups (A-K). Biomarker concentrations are in y-axis. Median shown as red horizontal line. *P* values were calculated by two-tailed unpaired t test or Mann-Whitney U test when appropriate. A- : amyloid negative, A+ : amyloid positive.

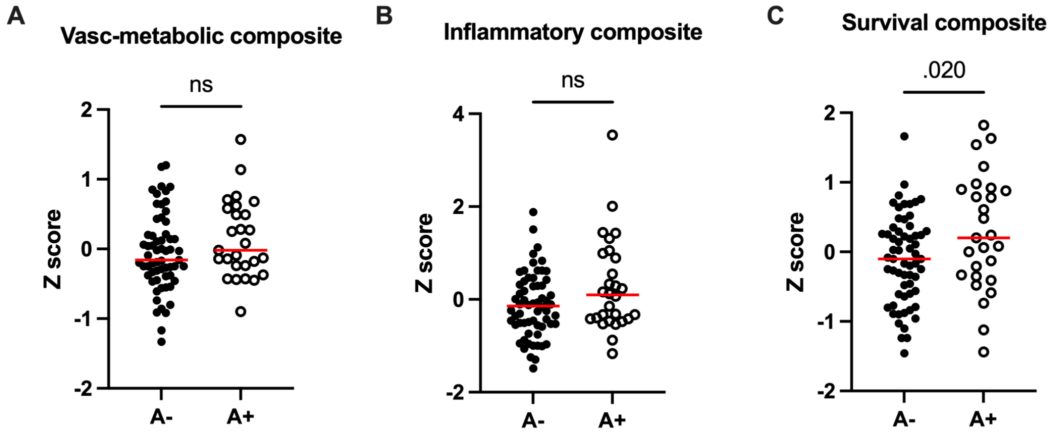

Supplementary Figure 3. Scatter-plots depicting of CSF biomarker composite scores across amyloid pathology status groups (A-K). Composite scores are in y-axis. Median shown as red horizontal line. *P* values were calculated by two-tailed unpaired t test or Mann-Whitney U test when appropriate. A- : amyloid negative, A+ : amyloid positive.

Supplementary Table 1. Basic characteristics of the amyloid status groups.

	A- (*n*=62)	A+ (*n*=28)	*P* value
Age, mean (SD)	64.4 (6.3)	67.6 (8.2)	0.054
Female, %	54.8	50	0.670
SCD/MCI/AD, %	48.4/29/22.6	0/42.9/57.1	<0.0001

P values were calculated by Mann-Whitney U test for age and by chi square for sex and diagnostic groups. A- : amyloid negative, A+ : amyloid positive

Supplementary Table 2. Sex differences in CSF biomarkers.

	**Men (*n*=62)**	**Women (*n*=28)**	***P* value**
SNAP-25	16.20 (2.70)	16.21 (2.37)	0.693
SYT-1	31.67 (7.52)	33.40 (7.52)	0.253
NG	207.15 (67.13)	236.98 (86.84)	**0.037**
NFL	1391.25 (682.82)	1286.00 (782.36)	0.290
IL-12/IL-23p40	5.10 (1.34)	5.78 (1.72)	**0.048**
IL-15	3.47 (0.85)	3.46 (0.84)	0.846
AGT	1332.44 (343.85)	1349.31 (403.45)	0.507
27-OH	1.43 (0.57)	1.26 (0.56)	0.294
TRX-1	4.84 (1.07)	4.86 (1.21)	0.999
ENPP-2	314.95 (36.06)	322.83 (38.17)	0.371
VEGF	4.03 (0.76)	3.94 (1.05)	0.334

Data are shown as mean (SD). *P* values were calculated by two-tailed unpaired t test or Mann-Whitney U as appropriate.

Supplementary Table 3. Human brain tissue donor characteristics.

**Demographics**	**Control (*n*=6)**	**AD (*n*=6)**	***P* value**
Age, mean (SD)	80.7 (7.3)	84.2 (6.3)	0.39
Female (%)	50 %	50 %	
PMI, mean (SD)	5:29 (0.04)	03:55 (0.07)	0.09
Braak Stage (0-VI)	0-II	V-VI	
